# Histopathological Features of SARS-CoV-2 in Extrapulmonary Organ Infection: A Systematic Review of Literature

**DOI:** 10.3390/pathogens11080867

**Published:** 2022-07-31

**Authors:** Diana Torge, Sara Bernardi, Mauro Arcangeli, Serena Bianchi

**Affiliations:** 1 Department of Life, Health and Environmental Sciences, University of L’Aquila, 67100 L’Aquila, Italy; diana.torge@graduate.univaq.it (D.T.); sara.bernardi@univaq.it (S.B.); mauro.arcangeli@univaq.it (M.A.); 2 Center of Microscopy, University of L’Aquila, 67100 L’Aquila, Italy

**Keywords:** COVID-19, SARS-CoV-2, histology, pathology, autopsy, post-mortem, biopsy

## Abstract

Severe acute respiratory syndrome coronavirus 2 (SARS-CoV-2) is a global health concern responsible for the ongoing pandemic. Histopathological pieces of evidence on COVID-19 are not fully investigated. This review aims to provide, through microscopy investigations, a histopathological overview of COVID-19 structural and ultrastructural alterations in different organs and tissues, excluding the respiratory system. The authors systematically reviewed the literature over the period February 2020–July 2022. Selected databases were PubMed, Scopus, and Google Scholar. The search strategy included the following terms: “COVID-19” or SARS-CoV-2 and “histopathology” or “pathology”; and “microscopy” and “liver”, “myocardium”,” spleen”, “testis”, and “placenta”. Preferred Reporting Items for Systematic Reviews and Meta-Analyses (PRISMA) guidelines were used. Thirty-one articles included in this systematic review demonstrated, at a histopathological level, that COVID-19 exerts detrimental effects on tissues, often promoting degenerative processes. Even if COVID-19 shows a histopathological tropism for the respiratory system, other tissues, from cardiovascular to reproductive, are affected by COVID-19. Therefore, this paper provides an up-to-date view of histopathological observations of the structural and ultrastructural alterations associated with COVID-19 and may contribute to a better knowledge of the physiopathological bases of this disease.

## 1. Introduction

Coronavirus disease 2019 (COVID-19) is a global health threat, commonly spread through respiratory droplets and aerosol transmission [1,2]. The COVID-19 pandemic started in the seafood market of Wuhan, China, in early December 2019 and then rapidly spread to Thailand, Japan, South Korea, Singapore, and Iran. After these initial months, the viral dissemination included Italy, Spain, the USA, the UAE, and the U.K. [3]. As a result, the World Health Organization (WHO) declared the outbreak of COVID-19 disease a pandemic on 11 March 2020 [3,4]. From this moment, the disease determined significant and forefront challenges: from virus isolation to vaccine development. The causative agent for COVID-19, severe acute respiratory syndrome coronavirus-2 (SARS-CoV-2), shows a genomic similarity of 82% with SARS-CoV-1, originated in China in 2002 and was isolated in the same year [5]. As regards the structure, it is an enveloped positive single-stranded RNA virus, with the most prominent viral genome of 8.4–12 kDa in size. There is a 5’ terminal in this viral genome, the central part of this genome, rich in open reading frames, which encodes proteins essential for virus replication. Instead, the 3’ terminal includes five structural proteins, Spike protein (S), membrane protein (M), nucleocapsid protein (N), envelope protein (E), and hemagglutinin-esterase protein (HE) [3,5]. The Spike protein is responsible for the pathogenesis in the human species since its receptor-binding domain (RBD) links to human cell surface receptor protein Angiotensin-Converting Enzyme-2 (ACE-2), encoded by the ACE2 gene. Hence, the virus, through the transmembrane protease serine 2 (TMPRSS2) [6], a cell–surface protein expressed by epithelial cells of specific tissues, is uploaded to the tissues [1].

ACE-2 has a ubiquitous distribution in the organs [7], with the consequence that SARS-CoV-2 infection may affect the lungs primarily, leading to respiratory failure [1]. However, this infection simultaneously involves several organs, from kidneys to the heart, blood vessels, liver, pancreas, and immune system [8]. Indeed, ACE-2 receptors are expressed in the digestive system, testis, and spleen. Furthermore, the entry of the virus into the host cells also enhances the immune response, with a deep secretion of inflammatory cytokines and chemokines. Therefore, SARS-CoV-2 can cause the production of multiple cytokines in body fluids, inducing acute respiratory distress and multiple organ failure [1]. In addition, other comorbidities affecting the vascular system, such as hypertension, diabetes, and renal failure, lead to a higher probability of respiratory failure and multiple organ failure [1]. As a forefront challenge is understanding the microscopic features of this multiorgan involvement, structural and ultrastructural evaluation of tissues derived from patients infected with SARS-CoV-2 might help in defining the underlying physiopathological dynamics of this infectious disease.

In this perspective, microscopy plays a crucial role, being a fundamental tool in clinical settings [9,10,11]. Light microscopy (LM) is an essential tool for describing the most significant morphological changes in tissues exposed to viral infection [12]. Transmission electron microscopy (TEM) and scanning electron microscopy (SEM) are powerful instruments in the investigation of intracellular and surface viral particles [13,14]. Confocal microscopy (CM) is a powerful adjunct to electron microscopy techniques in diagnostic and investigative virology, as it allows tissues analysis for evidence of viral infection at a cellular level [12]. Due to the underlying molecular dynamics of SARS-CoV-2 infection, its multiple effects on the physiology of several organs and tissues, and its histopathological and clinical outcomes, this manuscript aims to summarize the most recent histopathological evidence of tissues derived from COVID-19 patients and to understand the pathological features of this infection from a morphofunctional point of view. As covid-related histopathology of the respiratory system has already been extensively studied [15,16], we focused on damages in other body systems.

## 2. Results

**Figure 1:**
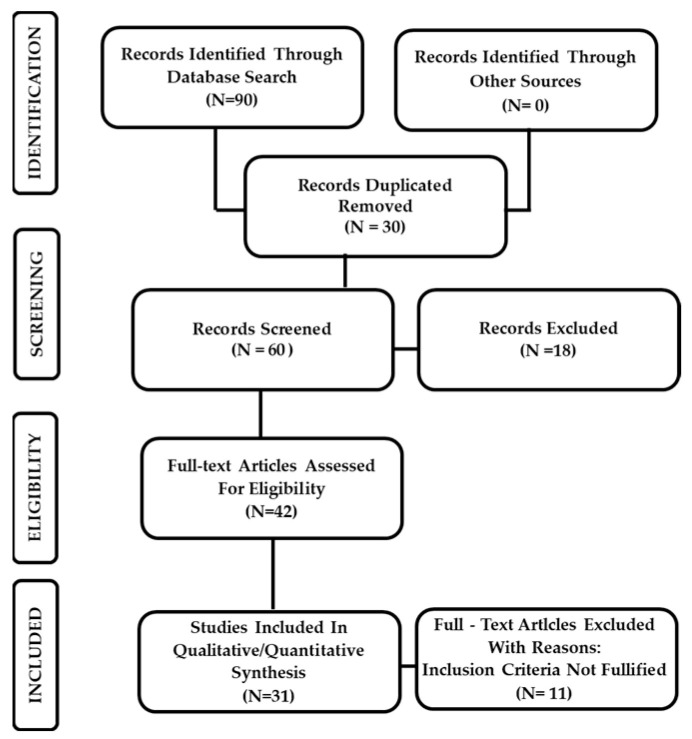
PRISMA flowchart illustrating the experimental study search and selection process.

Thirty-one studies published between February 2020 and July 2022 reported pathological findings, at autopsy or biopsy, obtained from patients with proven COVID-19 infection and met our inclusion criteria and were included in this study (Figure 1). These studies were classified according to the body system (Table 1) and to the microscopic techniques applied (Table 2).

### 2.1. Histopathological Evidence in the Cardiovascular System: Myocardium

Eight studies, published between 2020 and 2022, met our inclusion criteria and evaluated the most significant histopathological findings of COVID-19 in the cardiovascular system, specifically in the myocardium (Table 3).

#### 2.1.1. Histopathological Findings: Light Microscopy

**Figure 2:**
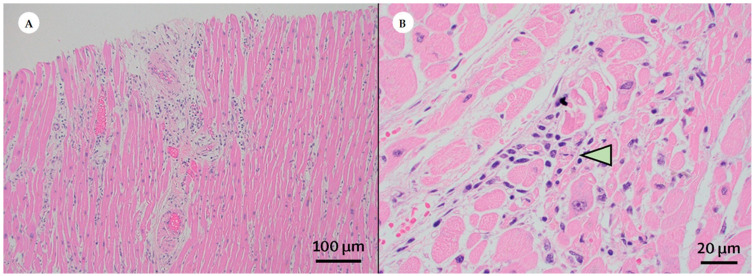
Histopathological findings in the cardiovascular system: the heart. (Courtesy: Bradley, B.T.; Maioli, H.; Johnston, R.; Chaudhry, I.; Fink, S.L.; Xu, H.; Najafian, B.; Deutsch, G.; Lacy, J.M.; Williams, T.; Yarid, N.; Marshall, D.A. Histopathology and Ultrastructural Findings of Fatal COVID-19 Infections in Washington State: A Case Series. Lancet 2020). (**A**) Light microscopy examinations on myocardial biopsy, derived from patients infected by SARS-CoV-2, reveal a significant myocyte damage. Haematoxylin and Eosin; Magnification: 40×. (**B**) By light microscopy, a considerable myocyte necrosis (arrowhead) is detected. Haematoxylin and Eosin; Magnification: 400×.

LM observations on myocardial biopsy showed significant myocyte necrosis (Figure 2A,B) [15,16] and focal, atypical myocyte degeneration [16]. Other morphological studies detected myocardial ischemic or inflammatory alterations, with pale and flabby myocardium and hypertrophy of myocytes [35]. Indeed, also mild perivascular lymphocytic infiltrate [37,38], but no cardiomyocyte alterations were found [37]. Additionally, Bois et al. found signs of active myocarditis, with focal active lymphocytic myocarditis [36]. Furthermore, Ramos-Rincon et al. report significant myocardial alterations as myocardial oedema, mononuclear interstitial infiltrate and necrosis of myocardial cells [28].

#### 2.1.2. Histopathological Findings: Transmission Electron Microscopy

Transmission electron microscopy analysis showed a significant cytopathy, with membrane damage and cytoplasmic vacuoles [25]; at the same time, single or small groups of viral particles were detected within the interstitial cells of the myocardium [25]. 

### 2.2. Histopathological Evidence in the Digestive System: Liver and Hepatocytes

Seven studies, published between 2020 and 2022, met our inclusion criteria and evaluated the most significant histopathological findings of COVID-19 in the digestive system, specifically in the liver and hepatocytes (Table 4).

#### 2.2.1. Histopathological Findings: Light Microscopy

LM examinations showed a significant cell degeneration, with the accumulation of nuclear glycogen in hepatocytes [29], diffuse parenchymal necrosis [35], and periportal lymphocytic inflammation [15]. Simultaneously, Fanni et al. detected the presence of enlarged hepatocytes with abundant granular cytoplasm [24]. Other morphological studies found slight to moderate hepatocyte swelling [45], sometimes apoptotic [30]. LM analysis also detected the presence of ballooning degeneration of hepatocytes [34]. Regarding liver sinusoids, Falasca et al. [35] and Fanni et al. [24] reported several alterations: sinusoidal congestion and dilatation, extravasation of red blood cells, presence of intrasinusoidal fibrillary aggregates and thrombi. Finally, Nava-Santana et al. detected significant neutrophilic sinusoidal inflammation and ballooning degeneration of hepatocytes [34].

#### 2.2.2. Histopathological Findings: Transmission and Scanning Electron Microscopy

TEM analysis revealed presence of vacuolar degeneration of hepatocytes, mitochondrial edema/swelling with disruption of cristae and expansion of endoplasmic reticulum [30,45]. Coronavirus particles were also detected in the cytoplasm of hepatocytes [30].

SEM examinations highlighted the presence of fibrillary aggregates of fibrin inside the sinusoidal lumen [24].

### 2.3. Histopathological Evidence in the Hematopoietic System: White and Red Pulp of the Spleen

Four studies, published between 2020 and 2022, met our inclusion criteria and evaluated the most significant histopathological findings of COVID-19 in the hematopoietic system, specifically the spleen (Table 5).

#### Histopathological Findings: Light Microscopy

Analysis conducted through an LM approach detected a significant depletion or atrophy [15,39] of splenic white pulp characterized by lymphoid hypoplasia [35] and lymphocytic depletion [40]. Regarding red pulp compartment, microscopic analysis revealed signs of congestion [35] and hemorrhage [39].

### 2.4. Histopathological Evidence in the Urinary System: The Kidney

Four studies, published in 2020, met our inclusion criteria and evaluated the most significant histopathological findings of COVID-19 in the urinary system, specifically in the kidney (Table 6).

#### 2.4.1. Histopathological Findings: Light Microscopy

Light microscopy examinations highlighted alteration of the functional unit of the kidney, the nephron. Commonly observed alterations were vacuolization of tubular epithelial cells [41], swelling [35], and hyperplasia [17] of glomerular endothelial cells, glomerular sclerosis, and diffuse parenchymal inflammation [15].

#### 2.4.2. Histopathological Findings: Transmission Electron Microscopy

Ultrastructural analysis confirmed the degeneration and the presence of vacuoles in tubular epithelial cells [41]. Viral particles were detected in both endothelial cells and proximal tubular epithelial cells [15,41]. Podocytopathy and glomerular endothelial inclusions were also revealed [17].

### 2.5. Histopathological Evidence in Endocrine System: Adrenal Glands

Three studies, published between 2020 and 2022, met our inclusion criteria and evaluated the most significant histopathological findings of COVID-19 in the endocrine system, specifically as regards the adrenal glands (Table 7).

#### Histopathological Findings: Light Microscopy

In samples of adrenal glands, LM observations revealed extensive areas of hemorrhagic necrosis, cortical lipid degeneration, and focal inflammation [42]. Furthermore, Paul et al. found inflammatory cell death and the presence of lymphocytes and histiocytes especially around the vessels [44]. Fitzek et al. reported a reduction of cytoplasmic lipid vacuoles [43]. 

### 2.6. Histopathological Evidence in Male Reproductive Tissue

Four studies, published between 2020 and 2022, met our inclusion criteria and evaluated the most significant histopathological findings of COVID-19 in the male genital system, specifically regarding testis and penis (Table 8).

#### 2.6.1. Histopathological Findings: Light Microscopy

**Figure 3:**
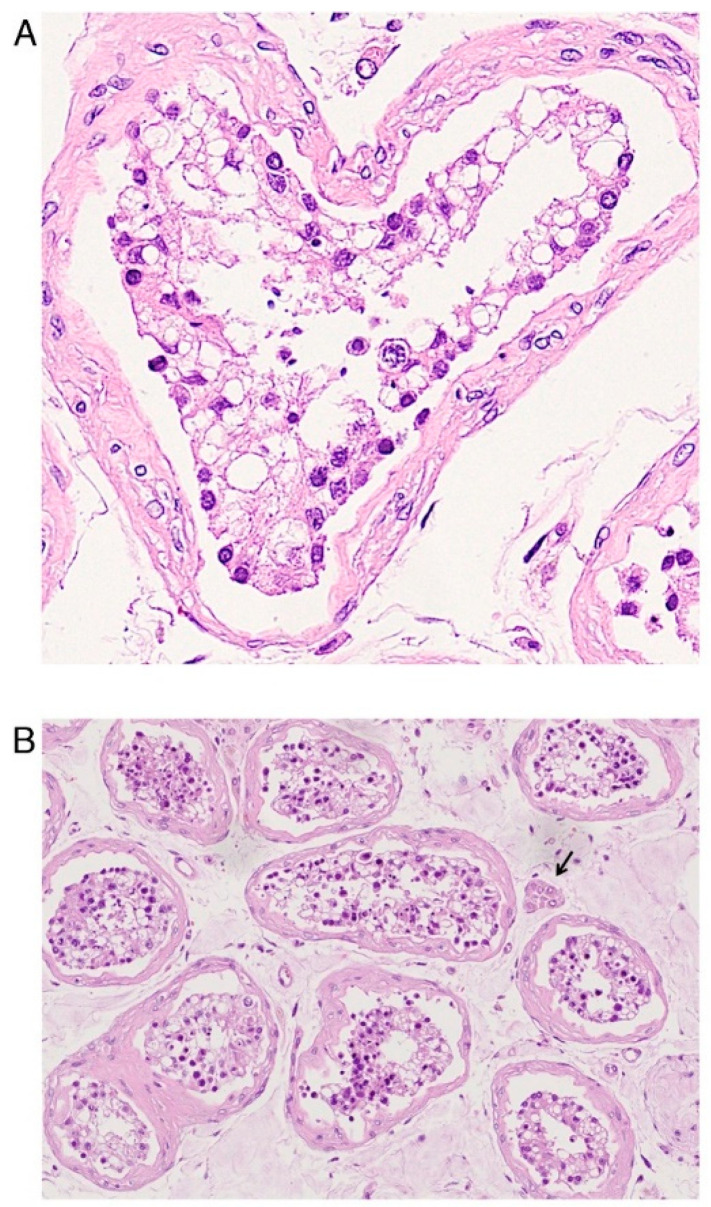
Histopathological findings in male reproductive tissue: testis. (Original labeling and Courtesy: Yang, M.; Chen, S.; Huang, B.; Zhong, J.M.; Su, H.; Chen, Y.J.; Cao, Q.; Ma, L.; He, J.; Li, X.F.; Li, X.; Zhou, J.J.; Fan, J.; Luo, D.J.; Chang, X.N.; Arkun, K.; Zhou, M.; Nie, X. Pathological Findings in the Testes of COVID-19 Patients: Clinical Implications. Eur. Urol. Focus 2020). (**A**) Defoliated and edematous Sertoli cells, characterized by vacuolation and cytoplasmic rarefaction. (**B**) Scattered Leydig cells (arrow). Hematoxylin and Eosin.

Morphological analysis of male reproductive tissue revealed alterations of Sertoli cells (Figure 3A), being edematous, vacuolated, and displaying cytoplasmic rarefaction, of interstitial Leydig cells (Figure 3B), and extensive germ cell destruction [31]. Moreover, hyalinization and thickening of the basement membrane of the seminiferous tubules with lymphocyte infiltration were also observed [32]. Ma et al. also reported selective degeneration of germ cells [33].

#### 2.6.2. Histopathological Findings: Transmission Electron Microscopy

Regarding testis, TEM examinations revealed the presence of coronavirus-like particles in the interstitial compartment [33] and inside the cytoplasm of interstitial cells [32]. Furthermore, extracellular viral particles, with prominent spikes and nucleocapsid, were found in the peri-vascular erectile tissue of penis [26].

### 2.7. Histopathological Evidence in Female Reproductive Tissue: Placenta

Four studies, published between 2020 and 2022, met our inclusion criteria and evaluated the most significant histopathological findings of COVID-19 in the female genital system, specifically regarding the placenta (Table 9).

#### 2.7.1. Histopathological Findings: Light Microscopy

Light microscopy examinations reported the presence of diffuse perivillous fibrin and inflammatory infiltrate, with macrophages and T lymphocytes, in placental tissues [18]. Placental intervillous hematomas, microvascular thrombosis, significant foci of decidual and villous inflammation were also observed [19]. Furthermore, Zaigham et al. detected a mixed inflammatory infiltrate, dominated by polymorphonuclear granulocytes, and a significant fibrinoid deposition in the intervillous space (intervillositis) [20].

#### 2.7.2. Histopathological Findings: Transmission Electron Microscopy 

TEM analysis demonstrated virus particles inside the cytosol of syncytiotrophoblast cells, cytotrophoblast cells, and fibroblasts [18,27].

### 2.8. Histopathological Evidence in the Integumentary System

Three studies, published in 2020 and 2021, met our inclusion criteria and evaluated the most significant histopathological findings of COVID-19 in the integumentary system (Table 10).

#### Histopathological Findings: Light Microscopy

Gianotti et al. found parakeratosis, acanthosis, dyskeratotic/necrotic keratinocytes, acantholytic clefts, and active lymphocyte satellitosis [21]. In addition, LM data also revealed the presence of scattered, necrotic, apoptotic keratinocytes, with smudging of the basement membrane [22], presence of superficial and deep infiltrate of lymphocytes, and vacuolar alteration of the basal layer of the epidermis [23].

## 3. Discussion

### 3.1. Microscopical Evidence in the Histopathology of the Myocardium

The spectrum of alterations in the myocardium is very wide (Table 3) including: myocyte necrosis (Figure 2A,B) [15,16], degeneration [16], destruction [28], hypertrophy [35] ischemic or inflammatory alterations [37], and active myocarditis [36]. From a clinical point of view, the most significant cardiovascular manifestations of COVID-19 are acute myocardial infarction, acute heart failure, cardiogenic shock, malignant arrhythmia, and myocarditis [46]. Undoubtedly, histopathological evaluation of cardiac tissue may help to understand COVID-19 pathophysiology and its clinical correlations [47,48].

Pathological alterations, which involve the heart tissue, may be associated with virus replication in the myocardium or, indirectly, with hypoxia or with an abnormal immune response determined by viral infection [46].

Hypoxia is a significant cause of acute cardiac injury in COVID-19 patients [49] and is obviously due to the diffuse alveolar damage caused by the direct viral cytopathic effects [50], but is also linked to systemic endothelial dysfunction and consequent massive microvascular immunothrombosis [51]. Several studies, in fact, detect a direct viral infection of endothelial cells, leading to a diffuse endothelial damage [52].

Numerous studies documented the presence of inflammatory infiltrate associated with COVID-19, but also ongoing heart inflammation after recovery from acute illness [50]. In this perspective, an immune cardiotoxicity, via cytokine-storm or by activating an autoimmune reaction against cardiac tissue, seems like a reasonable pathogenetic mechanism [50]. However, some studies on autopsies do not report the presence of lymphocytic infiltration; in this perspective, the immune-mediated cardiac injury does not appear to have a key role in these patients [53].

Undoubtedly, the reported alterations, such as myocyte necrosis and degeneration and the presence of viral particles in myocardial interstitial cells, but not in cardiomyocyte nor in endothelial cells [25], are perfectly in line with molecular evidence of SARS-CoV-2 particles localization in the interstitial cells [54]. These findings are consistent with prior reports, highlighting myocardial histopathological complications resulting from viral infection [55].

### 3.2. Microscopical Evidence in the Histopathology of Liver and Hepatocytes

Regarding liver, morphological data refers to hepatocytes and sinusoids (Table 4). Hepatocytes are a significant target of SARS-CoV-2 infection displaying cell degeneration, accumulation of nuclear glycogen, necrosis, enlargement, and swelling [15,29,30,35,45]. Additional morphological data highlighted the presence of apoptosis [30] and of ballooning degeneration [34]. Ultrastructural observations detected specific alteration of hepatocyte organelles as mitochondria and endoplasmic reticulum, vacuolar degeneration, and the presence of viral particles inside the cytoplasm [30,45].

Regarding liver sinusoids, structural and ultrastructural observation revealed congestion and extravasation of red blood cells [35], enlargement of sinusoids, and the presence of fibrin aggregates inside the sinusoidal lumen [24]. Inflammatory infiltrations were also observed, especially near the portal tracts [15,34,35].

From a clinical point of view, the occurrence of high transaminases blood levels in patients with severe COVID-19 is common [24] and there is a great debate on the mechanisms of hepatic involvement in SARS-CoV-2 infection. Is it the consequence of an immune-mediated phenomenon, of an ischemic injury, of medications (such as steroids), or the result of direct cytopathic damage [56,57]? 

Hepatocytes seem to be sensitive to SARS-CoV-2 infection because they constitutively express ACE2 [50] and structural and ultrastructural data confirm the direct hepatocellular injury [15,29,30,35,45]. Anyway, hepatic damage might be induced by other mechanisms. Indeed, COVID-19 is a prothrombotic disease [51], so congestion, extravasation of blood red cells, and presence of sinusoidal fibrin aggregates inside the vessels strongly support this hypothesis, and the occurrence of periportal inflammatory infiltrates may suggest another mechanism of liver injury, the endothelial-mediated inflammation [58].

### 3.3. Microscopical Evidence in the Histopathology of White and Red Pulp in the Spleen 

Regarding the structural changes of hematopoietic tissues during SARS-CoV-2 infection (Table 5), several studies revealed various degrees of depletion, atrophy, hypoplasia, and lymphocytic depletion of the white pulp of the spleen [15,35,39,40]. Regarding red pulp, morphological data revealed congestion and hemorrhages [35,39].

The depletion of white pulp compartment may be related to the compromised survival of lymphocytes caused by the virus [59], however, different mechanisms are under investigation, such as the direct organ attack by the virus, the microvascular dysfunction, or a cytokine-mediated pathogenesis [51,52,58,59,60]. In addition, molecular studies reported a considerable ACE2 receptors’ distribution in the red pulp sinus endothelium, sustaining morphological evidence [60].

### 3.4. Microscopical Evidence in the Histopathology of Tubular Epithelial and Glomerular Endothelial Cells

Significant histopathological findings regarding the kidney (Table 6) were found in the tubular epithelial cells, characterized by an extensive vacuolization and degeneration and containing abundant viral particles [15,41], but also, in glomerular compartment as the occurrence of glomerulosclerosis and alteration of glomerular endothelial and epithelial cells [15,17,35]. SARS-CoV-2 infection induces a significant impairment in renal function through the interaction with ACE2 receptors; undoubtedly, a detrimental role in this process is played by viral immune responses, cytokine storm, hypoxemia, and multiorgan dysfunction as previously described for other organs. It is universally recognized that ACE2 is widely expressed in human tubular epithelial cells [61], so kidney is affected by SARS-CoV-2 infection, principally via a direct cytopathic tubular injury [62]. However, the morphological data also report glomerular damage that seems to be less severe than tubular damage, but present. Beyond the endothelial or tubular pathogenetic mechanism, it is interesting to discuss the epithelial damage. In animal models, podocytes express the ACE2 receptor so the podocytopathy observed may be the result of virus direct cellular toxicity [63]. From this point of view, additional ultrastructural and molecular investigations are strongly required to determine the relationship between virus presence and podocyte damages.

### 3.5. Microscopical Evidence in the Histopathology of Endocrine System 

COVID-19 also exerts its viral tropism on endocrinological pathways, even if scant molecular and histopathological evidence exists on this topic. From a histopathological point of view, autopsy studies highlighted damaging of adrenal glands [42,43,44]. The perivascular inflammation, hemorrhage, and infarction probably are sustained by the associated inflammatory endotheliopathy, platelet dysfunction, and thrombosis [51,64] since endocrine glands are highly vascularized with numerous capillaries among the cells. The reduction of intracytoplasmic lipidic vacuoles deserves attention because it may reflect an alteration in cortisol dynamics. In severe cases of SARS-CoV-2 infection, the onset of a critical corticosteroid insufficiency is frequent [65]. This condition is characterized by an aberrant synthesis and secretion of cortisol, and a defective cellular corticosteroid activity [65]. ACE2 and TMPRSS2 proteins are expressed in the zona fasciculata/reticularis of the human adrenal cortex [65] suggesting the possibility of a direct cytopathic viral effect on these cells with consequence on hormonal synthesis and on body homeostatic processes.

### 3.6. Microscopical Evidence in the Histopathology of Male Reproductive Tissue 

SARS-CoV-2 infection exerts its histopathological effects also on male reproductive tissues (Table 8). In human testis, Sertoli cells (Figure 3A), interstitial (Leydig) cells (Figure 3B), and germ cells displayed several alterations as degeneration, enlargement, and vacuolization [31,33]. Seminiferous tubule basement membrane presented an inflammatory infiltrate, thickening and deposition of hyaline-like material [32]. Coronavirus-like particles were observed both in interstitial compartment of testis [32] and in the perivascular erectile tissue of the penis [26]. 

Recent molecular findings suggest that the direct cytopathic effect of SARS-CoV-2 in testicular cells is questionable because it requires the simultaneous expression of ACE and TMPRSS2 proteins [66]. Testicular cells display diffuse ACE2 distribution [67,68] but are deficient in TMPRSS2 expression [66]. Thus, the altered immunological and endothelial pathways seem to be the principal pathogenetic mechanisms sustaining impaired spermatogenesis observed in COVID-19 patients [51,66] together with the decrease of androgen production by adrenocortical steroidogenic cells [65].

Furthermore, endothelial integrity is essential for erectile function: for this reason, endothelial damage associated with COVID-19 is likely to compromise the penile vascular flow, determining an impairment in the erectile function [26,67]. Despite the few studies available, structural, and ultrastructural evidence on male reproductive tissues demonstrated how spermatogenic and erectile function may be irreversibly damaged by SARS-CoV-2 infection via immune or inflammatory pathways [68]. 

### 3.7. Microscopical Evidence in the Histopathology of Female Reproductive Tissue

Histopathological data from placental tissues (fibrin deposition and inflammatory infiltrate in the intervillous space) (Table 9) are suggestive of a severe inflammatory response, with a consequent impairment of the fetal–maternal barrier. From an ultrastructural point of view, the virus targeted placental cells seem to be trophoblasts and fibroblasts. TEM data showed virus particles within the cytosol of placental cells [18,27]. Syncytiotrophoblast cells cover the surface of the villous tree and are in contact with maternal blood, while fibroblasts are located inside villous core stroma between trophoblasts and fetal vessels [69].

The trophoblast coordinates the complex exchanges between the fetus and mother [69] so trophoblast degeneration and intervillositis can promote a progressive placental dysfunction and intrauterine demise [20]. Vascular alterations such as hematoma and thrombosis may represent structural counterpart leading to spontaneous abortion, fetal growth restriction, and severe early preeclampsia [20,70]. Morphological data suggest that different factors (direct cytopathy, ischemic injuries an inflammatory response) cooperate in compromising the physiological functions of the placenta as gas exchange, metabolic transfer, hormone secretion, and fetal protection [18].

### 3.8. Microscopical Evidence in the Histopathology of the Skin

Finally, the histopathological effects of SARS-CoV-2 infection on the integumentary system (Table 10) include interesting morphological changes involving both dermis and epidermis [21,22,23]. Skin alterations are super imposable to lesions commonly found in lupus erythematosus (the so-called chilblain lupus erythematosusllike eruption) [22,23], and are probably due to cytokine storm, especially to the interferon-I response [71], that promotes microvascular alterations, combining the immune and the thrombotic effects [23]. Moreover, keratinocytes have been proved to express ACE2 so are a potential target of a direct viral cytopathic effect [72].

### 3.9. Strengths and Limitation

This article provides a histopathological point of view on human body systems, with a wider overview of the infective outcomes of SARS-CoV-2. Obviously, given the novelty of the virus and the pandemic, few post-mortem studies have also focused on other organs. The difficulty in performing pre- and post-mortem biopsies and autopsies is also due to the high infectivity of SARS-CoV-2 and the uncertainty of safety, which endanger healthcare workers [73]. From this perspective, this systematic review relies on a small number of studies due to the poor use of autopsies and biopsies. Indeed, in a background of limited scientific knowledge and ongoing evidence on SARS-CoV-2 infection, autopsy and biopsy are crucial instruments to clarify COVID-19 pathophysiology and require specific operating procedures to minimize the risk of environmental contamination.

## 4. Materials and Methods

The current systematic review is registered on Open Science Framework database, with registration DOI: https://doi.org/10.17605/OSF.IO/B9GMV, accessed on 14 June 2022.

### 4.1. Search Strategy

This systematic review screened PubMed (www.ncbi.nlm.nih.gov, accessed on 14 June 2022), Scopus (https://www.scopus.com/standard/marketing.uri, accessed on 14 June 2022), and Google Scholar (Google Inc. Mountain View, CA, USA) databases to select high-profile studies. In addition, studies dealing with the virus were screened from February 2020 to July 2022. We adhered to the Preferred Reporting Items for Systematic Reviews and Meta-Analyses (PRISMA) guidelines [74].

### 4.2. Search Terms

The keywords used were: COVID-19 or “SARS-CoV-2” and “histopathology” or “pathology”; and “microscopy” and different terms related to the considered anatomical systems: “myocardium”, “endothelial cells”, “hepatocyte”, “liver sinusoid”, “spleen” and “white pulp” or “red pulp”, “tubular epithelial cells, “glomerular cells”, “reproductive system”, “endocrine system”, “keratinocyte”. This electronic search combined terms and descriptions linked to SARS-CoV-2 infection and pathological changes in different organs.

### 4.3. Inclusion and Exclusion Criteria

Articles (original, note, case series, case reports, brief report, correspondence, letter to editor, published) including pathological and histopathological findings of organs and tissues sampled from patients with COVID-19, during autopsy or through biopsy, from February 2020 to July 2022 were included in the study.

Studies met the following inclusion criteria: human studies; studies reporting pathologic findings at autopsy from patients with proven COVID-19 infection; studies reporting pathologic tissue findings of living biopsies obtained from proven COVID-19 patients; studies reporting pathologic tissue findings of post-mortem biopsies obtained from proven COVID-19 patients. Articles were excluded if they met the following exclusion criteria: studies on pediatric patients, narrative reviews, systematic reviews, articles not mentioning pathology and histopathology of organs, and studies reporting cases with incomplete information. As this is a systematic literature review, institutional ethical committee approval and informed consent are waived.

### 4.4. Study Selection

Two independent authors (S.B. and D.T.) dealt with the primary literature research. The same researchers conducted a second re-evaluation of the selected titles in which the studies that were not adapting to the established eligibility and inclusion criteria were deleted. Therefore, the remaining reports were intensely screened, considering the full-text articles for compatibility (Figure 1). In case of disagreements between the authors after independent evaluation, a consensus was reached by re-evaluation and discussion.

In the event of discrepancies in the data, reference paper authors were contacted by email for further explanation when possible. The remaining studies were finally reviewed for qualitative synthesis.

## 5. Conclusions

As COVID-19 is a global health concern, timely disease management is essential to fight this pandemic. For these reasons, the results presented by this systematic review provided structural (Figure 2 and Figure 3) and ultrastructural alterations associated with SARS-CoV-2 infection. These data, obtained through an LM, TEM, SEM, and CM approach, competitive methodologies in the field of microscopy, can be crucial not only in the future but also in the present to characterize the pathophysiology of SARS-CoV-2 infection, avoiding future health risks and providing, when combined to molecular evidence, forefront therapeutic strategy.

## Figures and Tables

**Table 1:** Studies included in the systematic review, classified according to the body system.

System	Number of Studies
Cardiovascular System	8
Digestive System	7
Lymphatic System	4
Urinary System	4
Endocrine System	3
Male Reproductive System	4
Female Reproductive System	4
Integumentary System	3

**Table 2:** Studies included in this systematic review, classified according to the methods of sampling and methods of microscopy used.

Methods of Sampling		Light Microscopy	Transmission Electron Microscopy	Scanning Electron Microscopy
	Methods of Microscopy			
Living Biopsy		Kudose et al. 2020 [17] Hosier et al. 2020 [18] Bertero et al. 2021 [19] Zaigham et al. 2022 [20] Gianotti et al. 2020 [21] Kolivras et al. 2020 [22] Sohier et al. 2021 [23] Fanni et al. 2021 [24]	Tavazzi et al. 2020 [25] Kresh et al. 2021 [26] Hosier et al. 2020 [18] Kudose et al. 2020 [17] Facchetti et al. 2020 [27]	Fanni et al. 2021 [24]
Post-mortem Biopsy		Ramos-Rincon et al. 2022 [28] Tian et al. 2020 [29] Wang et al. 2020 [30] Fanni et al. 2021 [24] Yang et al. 2020 [31] Achua et al. 2020 [32] Ma et al. 2021 [33] Nava-Santana et al. 2022 [34]	Wang et al. 2020 [30] Achua et al. 2020 [32] Ma et al. 2021 [33]	Fanni et al. 2021 [24]
Autopsy		Fox et al. 2020 [16] Bradley et al. 2020 [15] Falasca et al. 2020 [35] Bois et al. 2021 [36] Hartard et al. 2022 [37] Mezache et al. 2022 [38] Xiang et al. 2021 [39] González Pessolani et al. 2021 [40] Farkash et al. 2020 [41] Santana et al. 2020 [42] Fitzek et al. 2022 [43] Paul et al. 2022 [44]	Chu et al. 2021 [45] Farkash et al. 2020 [41] Bradley et al. 2020 [15]	

**Table 3:** Histopathological findings in the myocardium.

Author Year	Article Type	Tissue	Type of Sample	Microscopy	Histopathological Findings
Tavazzi et al. 2020 [25]	Case Report	Myocardium	Living Myocardial biopsy	Transmission Electron Microscopy	Cytopathy, with membrane damage and cytoplasmic vacuoles. Single or small groups of viral particles within the interstitial cells of the myocardium of the patient.
Fox et al. 2020 [16]	Original Research Article	Myocardium	Autopsy	Light Microscopy	Scattered individual cell myocyte necrosis. Cardiac myocytes showing focal, atypical myocyte degeneration.
Bradley et al. 2020 [15]	Original Research Article	Myocardium	Autopsy	Light Microscopy	Lymphocytic myocarditis. Necrotic myocytes.
Falasca et al. 2020 [35]	Original Research Article	Myocardium	Autopsy	Light Microscopy	Myocardial ischemic or inflammatory changes. Pale and flabby myocardium. Hypertrophy of myocytes.
Bois et al. 2021 [36]	Comparative Study	Myocardium	Autopsy	Light Microscopy	Active myocarditis, with focal active lymphocytic myocarditis.
Hartard et al. 2022 [37]	Case Report	Myocardium	Autopsy	Light Microscopy	Mild perivascular lymphocytic infiltrate, without cardiomyocytes alteration.
Mezache et al. 2022 [38]	Original Research Article	Myocardium	Autopsy	Light Microscopy	Scattered microvessels, enlarged, atypical endothelial cells. Microthrombi, perivascular extravasation of red blood cells. Perivascular edema and perivascular collections of mononuclear cells.
Ramos-Rincon et al. 2022 [28]	Original Research Article	Myocardium	Post-mortem biopsy	Light Microscopy	Myocardial edema. Mononuclear interstitial infiltrate. Necrotic myocardial cells.

**Table 4:** Histopathological findings in liver and hepatocytes.

Author Year	Article Type	Tissue	Type of Sample	Microscopy	Histopathological Findings
Tian et al. 2020 [29]	Original Research Article	Liver	Post-mortem biopsy	Light Microscopy	Hepatic cell degeneration. Nuclear glycogenation in hepatocytes. Focal necrosis.
Bradley et al. 2020 [15]	Original Research Article	Liver	Autopsy	Light Microscopy	Centrilobular necrosis. Mild periportal lymphocytic inflammation.
Wang et al. 2020 [30]	Original Research Article	Liver	Post-mortem biopsy	Light Microscopy	Apoptotic hepatocytes. Multinuclear syncytial hepatocytes. Vesicular steatosis. Lobular and portal tracts inflammation with lymphocytic infiltrate.
	Original Research Article	Hepatocytes	Post-mortem biopsy	Transmission Electron Microscopy	Viral particles in cytoplasm of hepatocytes. Enlarged mitochondria displaying obscure cristae and electron dense materials. Glycogen granule decrease.
Falasca et al. 2020 [35]	Original Research Article	Liver	Autopsy	Light Microscopy	Sinusoidal congestion. Extravasation of red blood cells. Hepatic necrosis. Inflammatory infiltration.
Chu et al. 2021 [45]	Original Research Article	Liver	Autopsy	Light Microscopy	Swelling of hepatocytes.
	Original Research Article	Hepatocytes	Autopsy	Transmission Electron Microscopy	Vacuolar degeneration of hepatocytes. Edema of mitochondria with disruption of cristae. Expansion of endoplasmic reticulum.
Fanni et al. 2021 [24]	Case Report	Liver	Post-mortem and living biopsy	Light Microscopy	Enlarged hepatocytes, with abundant granular cytoplasm, focally showing ground-glass appearance. Vesicular steatosis. Enlarged sinusoids, with abundant fibrillary aggregates. Sinusoidal dilatation, associated with scattered lymphocytes and red blood cells. Intrasinusoidal thrombi.
	Case Report	Liver	Post-mortem and living biopsy	Scanning Electron Microscopy	Fibrillary aggregates of fibrin inside the sinusoidal lumen.
Nava-Santana et al. 2022 [34]	Original Research Article	Liver	Post-mortem biopsy	Light Microscopy	Neutrophilic sinusoidal inflammation. Ballooning degeneration of hepatocytes.

**Table 5:** Histopathological findings in the white and red pulp of the spleen.

Author Year	Article Type	Tissue	Type of Sample	Microscopy	Histopathological Findings
Bradley et al. 2020 [15]	Original Research Article	White pulp	Autopsy	Light Microscopy	White pulp depletion.
Falasca et al. 2020 [35]	Original Research Article	White pulp Red pulp	Autopsy	Light Microscopy	Lymphoid hypoplasia in white pulp. Red pulp congestion.
Xiang et al. 2021 [39]	Original Research Article	White pulp Red pulp	Autopsy	Light Microscopy	White pulp atrophy. Congested and hemorrhagic red pulp.
González Pessolani et al. 2021 [40]	Brief Report	White pulp	Post-mortem biopsy	Light Microscopy	White pulp with lymphocytic depletion and diminished lymphoid follicles.

**Table 6:** Histopathological findings in the kidney.

Author Year	Article Type	Tissue	Type of Sample	Microscopy	Histopathological Findings
Bradley et al. 2020 [15]	Original Research Article	Kidney	Autopsy	Light Microscopy	Inflammation of the renal parenchyma. Focal segmental glomerulosclerosis. Tubular epithelial vacuolization.
	Original Research Article	Kidney	Autopsy	Transmission Electron Microscopy	Viral particles in endothelial cells and proximal tubular epithelial cells.
Farkash et al. 2020 [41]	Case Report	Tubular epithelial cells	Autopsy	Light Microscopy	Vacuolization of proximal tubular epithelial cells.
	Case Report	Tubular epithelial cells	Autopsy	Transmission Electron Microscopy	Vacuolization and degeneration of tubular epithelial cells, containing viral particles.
Kudose et al. 2020 [17]	Original Research Article	Kidney	Living biopsy	Light Microscopy	Hyperplasia of glomerular epithelial cells (podocytes). Collapse of glomerular capillaries.
	Original Re-search Article	Renal corpuscle	Living bi-opsy	Transmission Electron Microscopy	Podocytopathy. Glomerular endothelial inclusions.
Falasca et al. 2020 [35]	Original Research Article	Kidney	Autopsy	Light Microscopy	Swollen glomerular endothelial cells. Fibrin deposit under the Bowman capsule. Tubulointerstitial inflammation. Glomerular sclerosis.

**Table 7:** Histopathological findings in the adrenal glands.

Author Year	Article Type	Tissue	Type of Sample	Microscopy	Histopathological Findings
Santana et al. 2020 [42]	Case report	Adrenal glands	Autopsy	Light Microscopy	Extensive areas of hemorrhagic necrosis. Cortical lipid degeneration and focal inflammation.
Fitzek et al. 2022 [43]	Original Research Article	Adrenal glands	Autopsy	Light Microscopy	Reduction of cytoplasmic lipid vacuoles.
Paul et al. 2022 [44]	Original Research Article	Adrenal glands	Autopsy	Light Microscopy	Inflammation, with perivascular accentuation of small foci of lymphocytes and histiocytes. Inflammatory cell death.

**Table 8:** Histopathological findings in male reproductive tissue.

Author Year	Article Type	Tissue	Type of Sample	Microscopy	Histopathological Findings
Yang et al. 2020 [31]	Original Research Article	Testis	Post-mortem biopsy	Light Microscopy	Defoliated and edematous Sertoli cells. Scattered Leydig cells. Extensive germ cell destruction. Vacuolation and cytoplasmic rarefaction of Sertoli cells.
Achua et al. 2020 [32]	Original Research Article	Testis	Autopsy	Light Microscopy	Hyalinization and thickening of the basement membrane of the seminiferous tubules, with lymphocyte infiltration.
	Original Research Article	Testis	Autopsy	Transmission Electron Microscopy	Coronavirus-like particles in the cytoplasm of the interstitial cells of the testes.
Ma et al. 2021 [33]	Correspondence Article	Testis	Post-mortem biopsy	Light Microscopy	Degenerated germ cells sloughing into the lumen of the seminiferous tubules.
	Correspondence Article	Testis	Post-mortem biopsy	Transmission Electron Microscopy	Coronavirus-like particles in the interstitial compartment of testes.
Kresch et al. 2021 [26]	Original Research Article	Penis	Living Biopsy	Transmission Electron Microscopy	Extracellular viral particles in the peri-vascular erectile tissue.

**Table 9:** Histopathological findings in the placenta.

Author Year	Article Type	Tissue	Type of Sample	Microscopy	Histopathological Findings
Hosier et al. 2020 [18]	Case Report	Placenta	Living Biopsy	Light Microscopy	Diffuse perivillous fibrin and inflammatory infiltrate, with macrophages and T lymphocytes.
	Case Report	Placenta	Living Biopsy	Transmission Electron Microscopy	Virus particles in the cytosol of syncytiotrophoblast cells, cytotrophoblast cell and fibroblasts.
Facchetti et al. 2020 [27]	Original Research Article	Placenta	Living Biopsy	Transmission Electron Microscopy	Viral particles in the cytoplasm of syncytiotrophoblast cells and fibroblasts.
Bertero et al. 2021 [19]	Original Research Article	Placenta	Living Biopsy	Light Microscopy	Placental intervillous hematomas and microvascular thrombosis. Significant foci of decidual and villous inflammation.
Zaigham et al. 2022 [20]	Original Research Article	Placenta	Living Biopsy	Light Microscopy	Mixed inflammatory infiltrate in the intervillous space (intervillositis). Fibrinoid deposition in the intervillous space.

**Table 10:** Histopathological findings in the integumentary system.

Author Year	Article Type	Tissue	Type of Sample	Microscopy	Histopathological Findings
Gianotti et al. 2020 [21]	Letter to Editor	Skin	Living Biopsy	Light Microscopy	Parakeratosis, acanthosis, dyskeratotic keratinocytes, and small acantholytic cleft. Necrotic keratinocytes with lymphocyte satellitosis.
Kolivras et al. 2020 [22]	Case Report	Skin	Living Biopsy	Light Microscopy	Scattered, necrotic and apoptotic keratinocytes, with smudging of the basement membrane. Vacuolar alteration along the basal layer of the epidermis. Superficial and deep infiltrate of lymphocytes.
Sohier et al. 2021 [23]	Case Series	Skin	Living Biopsy	Light Microscopy	Superficial and deep infiltrate of lymphocytes. Vacuolar alteration of the basal layer of the epidermis.

## Data Availability

Data will be available upon reasonable request to the corresponding author.

## Abbreviations

SARS-CoV-2	Severe acute respiratory syndrome coronavirus 2
PRISMA	Preferred Reporting Items for Systematic Reviews and Meta-Analyses
COVID-19	Coronavirus disease 2019
WHO	World Health Organization
S	Spike protein
M	Membrane protein
N	Nucleocapsid protein
E	Envelope protein
HE	Hemagglutinin-esterase protein
RBD	Receptor-binding domain
ACE-2	Angiotensin converting enzyme 2
TMPRSS2	Transmembrane protease serine 2
LM	Light microscopy
TEM	Transmission electron microscopy
SEM	Scanning electron microscopy
CM	Confocal microscopy
